# Validation of the camouflaging autistic traits questionnaire short form (CATQ-SF)

**DOI:** 10.1016/j.comppsych.2024.152525

**Published:** 2024-08-22

**Authors:** Laura Hull, Will Mandy, Hannah Belcher, K.V. Petrides

**Affiliations:** a Department of Population Health Sciences, Bristol Medical School, University of Bristol, UK; b Research Department of Clinical, Educational & Health Psychology, University College London, UK; c Institute of Psychiatry, Psychology, and Neuroscience, King's College London, UK; d London Psychometric Laboratory, University College London, UK

**Keywords:** Autism, Camouflaging, Masking, Measure development

## Abstract

**Background:**

Camouflaging of autistic traits involves hiding or compensating for autistic characteristics, often due to stigma or a desire to fit in with others. This behaviour has been associated with mental health issues in autistic individuals. The 2 5-item Camouflaging Autistic Traits Questionnaire (CAT-Q) is the most commonly used self-report measure of camouflaging. In this study, a 9-item short form version was developed for use in clinical and research settings.

**Aims:**

To construct and psychometrically validate a brief self-report measure of camouflaging.

**Method:**

The Camouflaging Autistic Traits Questionnaire – Short Form (CATQ-SF) was developed and its factor structure and psychometric properties were evaluated in two studies. Study 1 used a large, online sample of autistic and non-autistic adults (*N* = 832) to evaluate the factor structure, psychometric properties, and measurement invariance of the CATQ-SF. Study 2 used an independent sample of autistic and non-autistic adults (*N* = 80) to test Study 1's findings.

**Results:**

In Study 1, evidence for a three-factor structure was observed, with good internal consistency (combined autistic & non-autistic α = 0.84). In addition, the instrument demonstrated measurement invariance, and reliably predicted higher levels of autistic traits. In Study 2, the 3-factor structure was replicated, and good internal consistency was again observed (combined autistic and non-autistic α = 0.89). In both studies, psychometric properties were of similar or higher validity compared to the full-form CAT-Q.

**Conclusions:**

The CATQ-SF can be used by clinicians and researchers to measure camouflaging in autistic and non-autistic adults quickly and reliably.

## 1 Introduction

The phenomenon of camouflaging of autism (which overlaps with closely related constructs such as masking, compensation, adaptive morphing, or passing) has received extensive research interest in the past decade [1,2]. Readers interested in the use of these terms should refer to recent commentaries [3] and reviews [2] for more detailed discussion of how camouflaging has been conceptualised and named. Camouflaging involves the hiding or compensating for autistic characteristics during social interactions, for instance by forcing oneself to make eye contact or hiding repetitive behaviours from others [4]. It can be a learned, practiced behaviour or an automatic response to experiencing stigma [5]. Camouflaging has been conceptualised as a subtype of impression management, the process of monitoring and modifying one's behaviour during social interactions [6]. However, there may be aspects of camouflaging that are distinctive to autism, including autism-specific motivations (such as avoiding autism-related stigma), neuro-cognitive processes (such as the role of executive functions), and consequences (including inauthenticity and impact on mental health). More research is needed to understand the potential overlap and distinctions between autistic camouflaging and typical impression management.

The most commonly used method of measuring camouflaging is the Camouflaging Autistic Traits Questionnaire (CAT-Q [7]), a 2 5-item self-report questionnaire comprising three subfactors that probe constructs compensation (the use of strategies to compensate for autism-related difficulties), masking (the hiding of autistic traits or using a non-autistic persona), and assimilation (the use of strategies to fit in with other people). Suitable for use in both clinical and non-clinical (i.e. non-autistic) populations [7], higher scores on the CAT-Q have been associated with poorer mental health outcomes including anxiety, depression, and suicidal ideation in both autistic and non-autistic populations [8–10]. A recent systematic review found that the CAT-Q had undergone the most extensive psychometric evaluation compared to other questionnaire-based methods of measuring camouflaging or similar constructs [11], although some areas of validity require further evaluation. The CAT-Q has been translated into multiple languages including French [12], Dutch [13], Mandarin Chinese [14], Italian [15], and Japanese [16].

The CAT-Q is widely used in clinical and research settings, however there are length limitations to its usefulness in the current format. A brief screening tool for camouflaging may be useful as part of autism diagnostic assessments. Concerns have been raised that some autistic individuals may not meet thresholds on screening tools due to having non-typical presentations of autism, including the use of camouflaging. These non-typical presentations are often not captured by current assessment tools – although we note that camouflaging should not be considered a prerequisite for an autism diagnosis [5,17].

Researchers seeking to measure camouflaging in relation to mental health, and to better understand the directional nature of this relationship [1], may also desire a shorter measure to be included in larger batteries of assessment. A short-form version of the CAT-Q would therefore allow inclusion in more studies, particularly population-based studies, and increase the measure's utility during clinical assessments.

This paper presents the development and validation of a 9-item version of the CAT-Q (CATQ-SF) in two different samples. Study 1 describes the construction and initial validation process in a ‘discovery sample’, which is the sample used to develop the original CAT-Q [7]. Study 2 describes the use of an independent sample of autistic and non-autistic British adults [18] as a ‘hold out sample’ to further validate the CATQ-SF.

## 2 Study 1

### 2.1 Methods

In Study 1, we tested a novel short-form version of the CAT-Q in the dataset used to develop the original CAT-Q [7].

#### 2.1.1 Participants

Participants were 832 adults (3 54 autistic, 478 non-autistic) recruited through an online survey. Participants were aged between 16 and 82 years (Mean = 36.01, SD = 14.84). See Hull et al. (2019) for full details of this sample. Written consent was obtained from all participants. The authors assert that all procedures contributing to this work comply with the ethical standards of the relevant national and institutional committees on human experimentation and with the Helsinki Declaration of 197 5, as revised in 2008. This study was approved by University College London Research Ethics Committee, project 747 5/002.

#### 2.1.2 Measures

Self-reported camouflaging was measured using the CAT-Q [7], a 2 5-item questionnaire with three factors measuring compensation, masking, and assimilation. Psychometric properties of the CAT-Q are described in the results section. In this paper, this measure will be referred to as the ‘full-form CAT-Q' to avoid confusion.

**Fig. 1:**
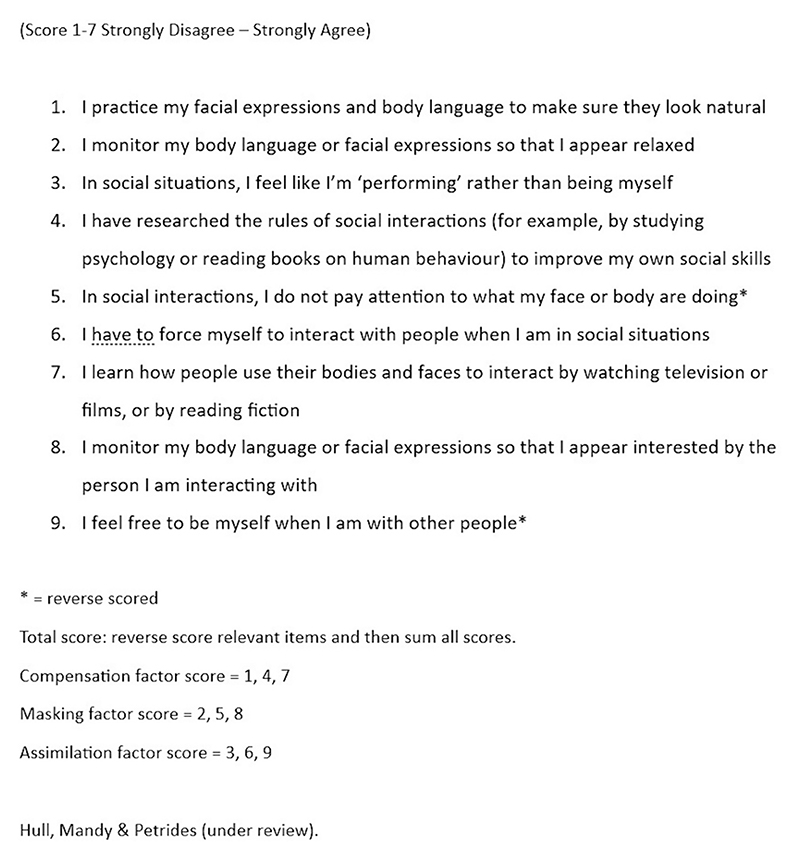
The Camouflaging Autistic Traits Questionnaire Short Form (CATQ-SF).

A 9-item short-form version of the CAT-Q was developed through discussion between three of the original CAT-Q authors (LH, WM and KVP), with three items representing each of the three factors. Items were selected based on a balance of 1) the highest loadings within each factor from the combined sample reported in Hull et al. (2019), and 2) the strongest conceptual link to each factor. The final version of the short form CAT-Q (CATQ-SF) is presented in Fig. 1.

Autistic-like traits were measured using the Broader Autism Phenotype Questionnaire (BAPQ [19]), a 36-item self-report measure. The BAPQ has demonstrated good internal consistency in this sample (α = 0.96).

#### 2.1.3 Analyses

We conducted a confirmatory factor analysis to test the three-factor model identified in the full-form CAT-Q. Diagonally Weighted Least Squares Means (WLSM) were used to account for the ordinal nature of the data [20]. Next, we compared mean scores between autistic and non-autistic groups. Convergent and criterion validity were evaluated respectively through correlations between the CATQ-SF and the full-form CAT-Q (with adjustment for part-whole overlap using Levy's correction [21]), and regression of autistic traits on the CATQ-SF. Finally, we evaluated measurement invariance across autistic and non-autistic groups.

Analyses for Study 1 were conducted separately in the autistic and non-autistic samples, and in the combined total sample, where appropriate. Results are presented alongside those from the full-form CAT-Q (data taken from Hull et al., 2019) to allow for comparison between the original and CATQ-SF. All analyses used the same statistical procedures and software (Rstudio, version 2023.06.2) as in the original paper by Hull et al., (2019). Full code for analyses is available from the corresponding author upon request.

### 2.2 Results

**Fig. 2:**
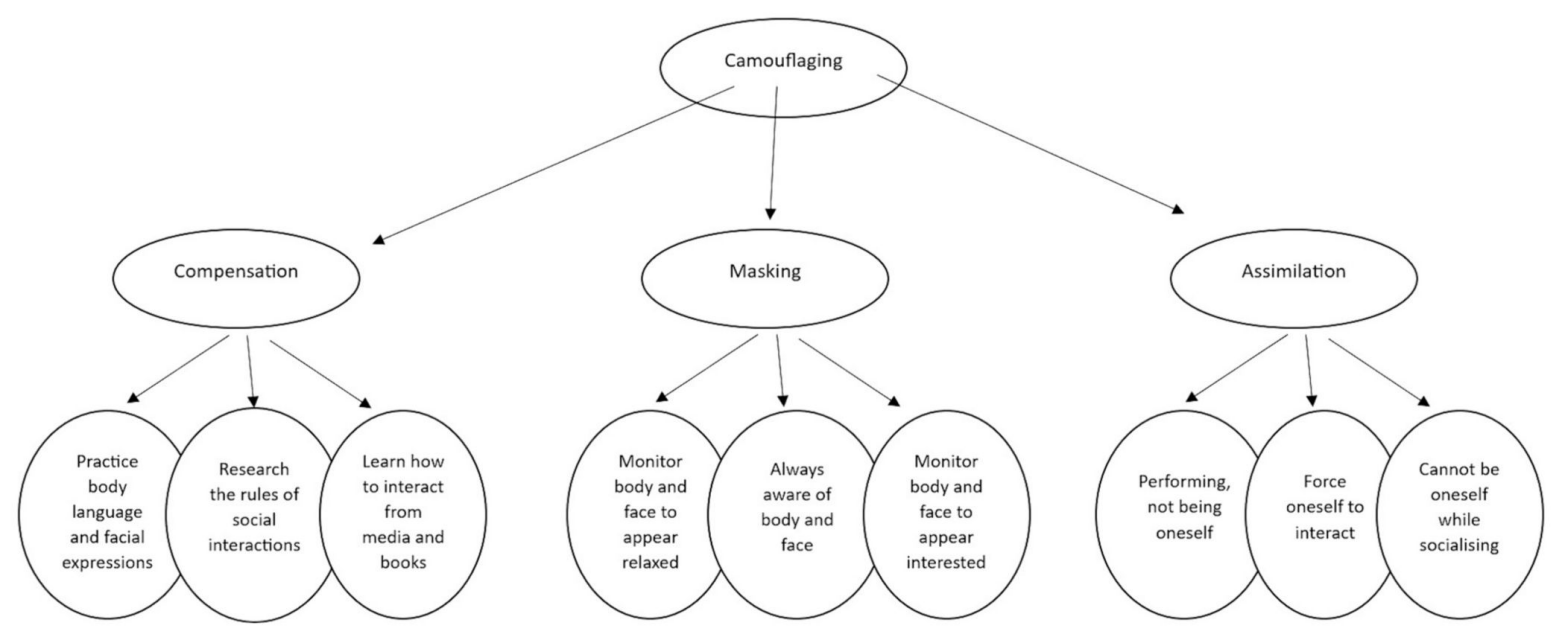
Conceptual model of the CATQ-SF.

Results of confirmatory factor analysis of the original 2 5-item model and the 9-item model are reported in Table 1, for the autistic, non-autistic, and combined samples. A three-factor model (see Fig. 2) was found to provide an acceptable fit. Overall, the fit indices were comparable between the original and the short-form versions: Comparative Fit Index (CFI) values exceeded 0.9 5, and RMSEA and SRMR values were below 0.06 and 0.08, respectively, in all samples. Cronbach's alphas were comparable across both original and short-form versions in all samples, indicating acceptable levels of internal consistency (α >0.70).

Measurement invariance was demonstrated at the metric and scalar level between autistic and non-autistic individuals, but was not upheld at the residual level (Table 2), which returned a CFI change greater than 0.01.

Autistic participants scored higher than non-autistic participants on the total CATQ-SF and all its subscales, although only a small difference was observed for the masking subscale, in keeping with the corresponding findings on the full-form CAT-Q (Table 3).

Across the autistic, non-autistic, and combined samples, the full-form and short-form CAT-Q were strongly correlated at both total and subscale levels (Tables 4a and 4b). The CATQ-SF and its subscales were correlated with autistic traits in the non-autistic and combined samples, but its compensation and masking subscales were not correlated with the BAPQ in the autistic sample. This mostly replicates associations between the full-form CAT-Q and the BAPQ, with the exception of the autistic sample, where the compensation subscale was positively associated with autistic traits, unlike for CATQ-SF compensation. Overall, 88% of correlations with external criteria reached statistical significance for both the full and short forms of the CAT-Q.

Finally, these associations were corroborated by the regression analyses (Table 5), which showed that the total CATQ-SF and all its subscales predicted autistic traits in both the non-autistic and combined samples. However, the compensation and masking subscales did not predict autistic traits in the autistic sample.

### 2.3 Discussion

We tested whether the CATQ-SF followed the same three-factor structure as the full-form CAT-Q in the original dataset. Confirmatory factor analysis revealed the three-factor model to be a better fit than the full-scale CAT-Q, due to the reduced psychometric complexity of the short form and inclusion of only the best-fitting items. The CATQ-SF also showed good internal consistency for the total scale and subscales in the autistic, non-autistic, and combined samples. However, the internal consistency of the CATQ-SF compensation subscale was lower, while still acceptable, in the autistic sample.

Measurement invariance between autistic and non-autistic individuals was observed at the configural, metric, and scalar levels, suggesting that the CATQ-SF has similar factor loading, item loading, and item intercepts in both these groups. However, residual invariance was not supported in this study. This contrasts with the full-form CAT-Q, which demonstrated full measurement invariance between autistic and non-autistic males and females in its original validation (Hull et al., 2019).

Residual invariance is not a prerequisite for mean comparisons [22]. Therefore, we argue for the CATQ-SF being suitable for comparing autistic and non-autistic groups. In addition, the acceptable model fit in both autistic and non-autistic samples demonstrates that the CATQ-SF is appropriate for use in both groups. We do, however, acknowledge that evidence of measurement non-invariance, and alternative factor structures, has been reported in translated versions of the full-scale CAT-Q [13]. This suggests that there may be cultural factors impacting performance and factor structure in the full-scale CAT-Q.

Autistic participants scored higher on average than non-autistic participants on the CATQ-SF, replicating group differences obtained with the full-form CAT-Q [1]. As further evidence for criterion validity, CATQ-SF total and subscale scores predicted autistic traits in the non-autistic and combined samples. Only the assimilation subscale predicted autistic traits in the autistic sample. This suggests that for autistic individuals, the CATQ-SF may capture more aspects of camouflaging associated with stigma or appearing different [5], rather than camouflaging of autistic traits more generally as seen in the combined sample. In contrast to the full-scale CAT-Q, the compensation subscale of the CATQ-SF did not predict autistic traits in the autistic sample. The items included in the short-form compensation subscale may be less strongly related to autistic traits as measured by the BAPQ. Future research should compare the CATQ-SF subscales to other measures of autistic traits to further test construct validity.

## 3 Study 2

### 3.1 Methods

Study 2 evaluated the CATQ-SF in a novel sample of British autistic and non-autistic adults, using data from Belcher et al. (2022).

#### 3.1.1 Participants

Participants were 80 adults (40 autistic, 40 non-autistic) recruited through advertisements on social media and local universities. Participants were aged between 18 and 40 years (Mean = 26.71, SD = 6.43). See Belcher et al. (2022) for full details of this sample. Written consent was obtained from all participants. The authors assert that all procedures contributing to this work comply with the ethical standards of the relevant national and institutional committees on human experimentation and with the Helsinki Declaration of 197 5, as revised in 2008. All procedures involving human subjects/patients were approved by Anglia Ruskin University Ethics Committee, reference EHPGR-11.

#### 3.1.2 Measures

The full-form CAT-Q was administered to all participants. Using these data, CATQ-SF scores were calculated as described above. Psychometric properties for both versions of the measure are described in the results section.

Autistic traits were assessed using the Autism Quotient (AQ [23]), which demonstrated acceptable internal consistency in this sample (α = 0.67).

#### 3.1.3 Analyses

Confirmatory factor analysis, mean score comparison, and associations with autistic traits (AQ) were evaluated, in the combined autistic and non-autistic sample, as above.

### 3.2 Results

Confirmatory factor analysis indicated an acceptable model fit for both the full- and short-forms of the CAT-Q (Table 6), with the CATQ-SF demonstrating a better fit according to lower RMSEA and SRMR values. This aligns with expectations, given the smaller number of items and lower psychometric complexity of the reduced form of the instrument [24]. Internal consistency was good at both levels (total and subscale) of both CATQ versions.

Autistic participants scored higher than non-autistic peers on the total CAT-Q (original and short form) and all subscales except masking (Table 7).

Moderate-to-strong Levy-corrected correlations were obtained between the full-and short-form CAT-Q in this sample. Patterns of association with autistic traits were the same across both versions, with the total and all subscale scores demonstrating a positive correlation with the AQ. The sole exception concerned the masking subscale, which was not associated with the AQ for either full- or short-form (Table 8).

These associations were reflected in the subsequent regression models, where both the total and the subscale scores of the CAT-Q and CATQ-SF predicted greater levels of autistic traits. The masking subscale was again the sole exception in both versions of the instrument (Table 9).

### 3.3 Discussion

In this smaller, novel, sample, the three-factor structure of the CATQ-SF was supported, and was again found to provide a better fit than for the full-form. Internal consistency for the total CATQ-SF and subscale scores was good. Relatively large group differences were observed between autistic and non-autistic participants, suggesting that the measure captures behaviours that are generally more common in autistic people.

The total and subscale scores of both the full and short forms of the CAT-Q were significantly correlated with each other. A similar pattern of associations with autistic traits was observed for both versions of the CAT-Q. Both the compensation and assimilation subscales predicted autistic traits, but the masking subscale did not in either version of the CAT-Q. Overall this suggests that the CATQ-SF has similar convergent and criterion validity as the full-form CAT-Q.

## 4 General discussion

This study evaluated the psychometric properties of a new short-form version of the Camouflaging Autistic Traits Questionnaire in two samples: a large online sample used to construct the original CAT-Q, and a smaller novel sample of British adults. In both samples, evidence for a three-factor structure of the CATQ-SF was found. Model fit for the CATQ-SF was good in both samples in this study, with acceptable internal consistency across samples and diagnostic groups at both the total and subscale level. A three-factor model, comprising the subscales of compensation, masking, and assimilation, is appropriate for the CATQ-SF to capture a range of different camouflaging behaviours. Measurement invariance was also demonstrated between autistic and non-autistic participants in Study 1. Additionally, we obtained evidence of criterion validity in the form of mean level differences between autistic and non-autistic participants, and significant associations with autistic traits.

Previous studies using the full-scale CAT-Q have found that autistic adults tend to score higher than non-autistic adults [1,7], although group differences on the Masking subscale are not always observed [25]. Our findings confirmed this trend; autistic adults recorded higher scores than their non-autistic peers on the total CATQ-SF and its Compensation and Assimilation subscales in both samples. Additionally, in the larger sample, autistic adults also scored higher on the Masking subscale. The group differences observed ranged from moderate to large effects, which is consistent with the effect sizes reported in other studies [1].

In this study, strong correlations between the full- and short-form versions of the total score, and each subscale, were observed in both combined samples, and in the autistic and non-autistic subsamples in Study 1. This suggests that the CATQ-SF is conceptually similar to the corresponding full-form, capturing a comparable range of variance in scores across different samples.

Camouflaging, as measured using the CAT-Q and other methods, has been associated with higher autistic traits in both autistic and non-autistic populations [26,27]. In both samples in the present study, higher CATQ-SF subscale scores predicted greater levels of autistic traits overall. There was only the exception of the masking and compensation subscales for autistic adults in Study 1, and the masking subscale in Study 2. The masking component of the CAT-Q has previously been proposed as the least distinctive between autistic and non-autistic individuals. Masking represents responses to experiencing stigma due to appearing autistic, leading to the concealment of features associated with perceived difference [2]. Similar patterns of association were found between the full- and short-form CAT-Q, confirming the robustness of the relationship. The consistency of this finding suggests that the masking subscale may capture aspects of camouflaging driven by experiencing stigma and potentially a minority identity, rather than being unique to autistic experiences [28]. Future research would benefit from exploring how masking (and the CATQ more broadly) varies across individuals with different stigmatised identities, including mental health conditions such as social anxiety, and racial and ethnic minority identities.

The compensation subscale predicted autistic traits for participants in Study 2, but not for autistic participants in Study 1. One potential explanation for this is the different measures used to capture autistic traits; the BAPQ was used in Study 1 and the AQ in Study 2. It may be that for autistic adults, compensation is related to only certain autistic traits and thus the relationship may vary depending on how autistic traits are measured. An alternative explanation is that the relationship between compensation and autistic traits is stronger for non-autistic individuals, and therefore was significant in the combined sample in Study 2 but not in the autistic subsample in Study 1. More research in larger subsamples of autistic and non-autistic individuals is needed to confirm these findings.

### 4.1 Strengths and limitations

Strengths of this study include the combination of a large discovery sample for initial validation and a smaller, hold-out, sample for replication of psychometric evaluations. Another strength was the use of two different measures of autistic traits, demonstrating criterion validity at the conceptual level rather than with one specific measure. However, a key limitation of both samples used here is that the diagnostic status of participants was not independently assessed. Accordingly, we cannot verify that all self-reported autistic adults would meet diagnostic criteria for autism. In addition, the second sample used for validation was relatively small, and therefore more precise sub-sample evaluations, such as measurement invariance between groups, could not be performed. We recommend further evaluation of the CATQ-SF in larger clinical samples, to allow for more rigorous investigation of group differences as well as replication in other cultures, since participants in the present study were predominantly British.

### 4.2 Recommendations for use

Overall, our studies suggest that the CATQ-SF is a valid and reliable measure of camouflaging behaviours, and is broadly comparable to the full-scale CAT-Q. We recommend that researchers or practitioners who are seeking a brief and easily administered measure of camouflaging use the CATQ-SF, reporting both total and subscale scores. However, we note that due to the limitations described above, the CATQ-SF may not always be the optimal choice. For instance, if researchers are interested in exploring the relationship between specific subscales and autistic traits, the full-scale CAT-Q may be a more appropriate measure to use as it may contain more items that are relevant to autistic traits. The full-scale CAT-Q will also, by nature of having more items, allow for greater variation to be captured both across and within groups, than the CATQ-SF. We therefore advocate for the use of the full-scale CATQ where possible, and the CATQ-SF as an appropriate alternative when there are space or time constraints.

## Figures and Tables

**Table 1:** Confirmatory Factor Analysis of Three-factor Model Using Autistic, Non-Autistic, and Combined samples from Hull et al. (2019).

Autistic (*N* = 3 54)	Items	Chi square	CFI	RMSEA (9 5% CI)	SRMR	Cronbach's alpha
**Full-form CAT-Q (Total)**	25	χ^2^ = 596.9 5, *p* < .001	0.97	0.056 (0.050–0.063)	0.07 5	0.91
Compensation	9					0.88
Masking	8					0.87
Assimilation	8					0.86
**CAT-Q SF**	9	χ^2^ = 49.33, *p* = .002	0.98	0.05 5 (0.033–0.077)	0.060	0.78
Compensation	3					0.62
Masking	3					0.76
Assimilation	3					0.8 5
**Non-autistic *(N =* 478)**	**Items**	**Chi square**	**CFI**	**RMSEA (9 5% CI)**	**SRMR**	**Cronbach's alpha**
**Full-form CAT-Q (Total)**	25	χ^2^ = 619.10, p < .001	0.98	0.046 (0.041–0.051)	0.058	0.93
Compensation	9					0.90
Masking	8					0.84
Assimilation	8					0.89
**CAT-Q SF**	9	χ^2^ = 38.7 5, *p* = .029	0.99	0.037 (0.000–0.072)	0.028	0.8 5
Compensation	3					0.73
Masking	3					0.73
Assimilation	3					0.74
**Combined (N = 832)**	**Items**	**Chi square**	**CFI**	**RMSEA (9 5% CI)**	**SRMR**	**Cronbach's alpha**
**Full-form CAT-Q (Total)**	25	χ^2^ = 969.53, p < .001	0.98	0.052 (0.048–0.05 5)	0.057	0.94
Compensation	9					0.92
Masking	8					0.86
Assimilation	8					0.93
**CAT-Q SF**	9	χ^2^ = 53.12, *p* = .001	0.99	0.039 (0.02 5–0.053)	0.040	0.84
Compensation	3					0.74
Masking	3					0.76
Assimilation	3					0.86

**Table 2:** Multi-Group Measurement Invariance in CAT-Q Short-Form (Autistic *N* = 354, Non-Autistic N = 478) in the Combined Sample from Hull et al. (2019).

Model	χ^2^	ΔX^2^	Df	CFI	ΔCFI
1. Configural invariance	83.27	–	48	1.00	–
2. Metric invariance	10 5.69	22.42	54	1.00	0
3. Scalar invariance	152.6 5	46.96	60	0.991	0.008
4. Residual invariance	228.9 5	76.3	69	0.97 5	0.016

**Table 3:** Mean scores and Differences for Autistic and Non-Autistic Groups from Hull et al. (2019).

	Mean score (Non-autistic)	Mean score (Autistic)	Difference between groups	Effect size (Cohen’s d)
**Full-form CAT-Q (Total)**	3.48	4.79	*t =* 12.98, p < .001	d = 0.67
Compensation	2.89	4.42	*t =* 11.90, p < .001	d = 0.87
Masking	4.29	4.5 5	*t =* 2.19, *p* = .03	d = 0.13
Assimilation	3.32	5.29	*t =* 16.3 5, *p*< .001	d = 1.03
**CAT-Q SF (Total)**	3.33	4.63	*t =* 12.92, p < .001	d = 1.14
Compensation	2.51	4.30	*t =* 14.16, p < .001	d = 1.24
Masking	4.09	4.43	*t =* 3.22, p *=* .001	d = 0.28
Assimilation	3.40	5.10	*t =* 13.12, p < .001	d = 1.13

Note: Mean scores have been rescaled to vary between 1 and 7. CAT-Q = Camouflaging Autistic Traits Questionnaire. SF = Short Form.

**Table 4a:** Intercorrelation Matrix for the Autistic (above Diagonal) and Non-Autistic (below Diagonal) Samples from Hull et al. (2019).

	FF CAT-Q Total	FF Comp	FF Mask	FF Assim	CAT-Q SF	SF Comp	SF Mask	SF Assim	BAPQ
FF CAT-Q Total		0.8 5*	0.78*	0.72*	0.8 5*	0.67*	0.6 5*	0.61*	0.36*
FF Comp	0.88*		0.50*	0.50*	0.81*	0.83*	0.51*	0.4 5*	0.24*
FF Mask	0.79*	0.56*		0.29*	0.79*	0.52*	0.8 5*	0.34*	−0.04
FF Assim	0.87*	0.63*	0.52*		0.64*	0.37*	0.29*	0.83*	0.73*
CAT-Q SF	0.86*	0.84*	0.77*	0.83*		0.73*	0.74*	0.59*	0.29*
SF Comp	0.73*	0.8 5*	0.50*	0.56*	0.87*		0.54*	0.3 5**	0.10
SF Mask	0.70*	0.59*	0.8 5*	0.51*	0.7 5*	0.53*		0.34*	0.01
SF Assim	0.7 5*	0.58*	0.94*	0.8*	0.76*	0.51*	0.49*		0.66*
BAPQ	0.69*	0.54*	0.36*	0.81*	0.6 5*	0.47*	0.40*	0.74*	

* *p* < .001. Note: CATQ = Camouflaging Autistic Traits Questionnaire; FF = Full Form; SF = Short Form; Comp = Compensation subscale; Mask = Masking subscale; Assim = Assimilation subscale; BAPQ = Broader Autism Phenotype Questionnaire.

**Table 4b:** Intercorrelation Matrix for the Combined Sample from Hull et al. (2019).

	FF CAT-Q Total	FF Comp	FF Mask	FF Assim	CAT-Q SF	SF Comp	SF Mask	SF Assim
FF Comp	0.089*							
FF Mask	0.71*	0.49*						
FF Assim	0.8 5*	0.67*	0.38*					
CAT-Q SF	0.7 5*	0.86*	0.71*	0.80*				
SF Comp	0.60*	0.71*	0.47*	0.59*	0.86*			
SF Mask	0.49*	0.53*	0.70*	0.40*	0.77*	0.52*		
SF Assim	0.60*	0.63*	0.38*	0.73*	0.81*	0.56*	0.40*	
BAPQ	0.67*	0.57*	0.16*	0.83*	0.63*	0.49*	0.21*	0.79**

* p < .001. Note: CAT-Q = Camouflaging Autistic Traits Questionnaire; FF = Full Form; SF = Short Form; Comp = Compensation subscale; Mask = Masking subscale; Assim = Assimilation subscale; BAPQ = Broader Autism Phenotype Questionnaire.

**Table 5:** Models regressing the BAPQ on the Full-Form (FF) and Short-Form (SF) CAT-Q Subscales in the Autistic, Non-Autistic, and Combined Sample from Hull et al. (2019).

		Autistic					Non-autistic					Combined			
Predictor		R^2^ Adj	F(df)	B	p		R^2^ Adj	F(df)	B	p		R^2^ Adj	F(df)	B	p
FF Compensation		0.0 5	17.53 (1,299)	0.50	<0.001		0.29	186.9 (1,444)	1.26	<0.001		0.32	357.2 (1,745)	1.4 5	<0.001
FF Masking		−0.01	0.51 (1,299)	−0.10	0.476		0.13	66.78 (1,444)	1.11	<0.001		0.02	19.29 (1,745)	0.54	<0.001
FF Assimilation		0.54	347.6 (1,299)	1.97	<0.001		0.6 5	812.1 (1,444)	1.89	<0.001		0.70	170 5 (1,745)	2.28	<0.001
SF Compensation		0.01	3.15 (1,299)	0.5 5	0.077		0.23	130.7 (1,444)	2.91	<0.001		0.24	234.9 (1,745)	3.28	<0.001
SF Masking		−0.01	0.01 (1,299)	0.02	0.93 5		0.13	70.33 (1,444)	2.40	<0.001		0.04	34.99 (1.745)	1.56	<0.001
SF Assimilation		0.5 5	239.6 (1,299)	4.30	<0.001		0.5 5	534.4 (1,444)	4.28	<0.001		0.63	1277 (1,745)	5.24	<0.001

**Table 6:** Confirmatory Factor Analysis of the Three-Factor Model Using the Combined Sample from Belcher et al. (2022).

	Items	Chi square	CFI	RMSEA (9 5% CI)	SRMR	Cronbach's alpha
**Original CAT-Q (Total)**	25	χ^2^ = 220.98, *p* = .99	1.00	0.070 (0.061−0.079)	0.096	0.94
Compensation	9					0.90
Masking	8					0.83
Assimilation	8					0.92
**CAT-Q SF**	9	χ^2^ = 5.77, p = .99	1.00	0.001 (0.001−0.001)	0.039	0.89
Compensation	3					0.83
Masking	3					0.87
Assimilation	3					0.81

**Table 7:** Mean Scores and Differences for Autistic and Non-Autistic Groups from Belcher et al. (2022).

	Mean score (Non-autistic)	Mean score (Autistic)	Difference between groups	Effect size (Cohen's d)
**Original CAT-Q (Total)**	3.54	4.40	*t =* 4.93, p < .001	d = 1.08
Compensation	2.88	4.56	*t =* 5.7 5, p < .001	d *=* 1.29
Masking	4.4 5	4.61	*t =* 0.58, *p* = .57	d *=* 0.13
Assimilation	3.49	5.14	*t =* 5.6 5, p < .001	d *=* 1.26
**CAT-Q SF**	3.34	4.58	*t =* 4.09, p < .001	d *=* 0.92
Compensation	2.33	4.06	*t =* 4.34, p < .001	d *=* 1.04
Masking	4.02	3.52	*t =* 0.79, *p* = .43	d *=* 0.18
Assimilation	3.49	5.15	*t =* 5.23, p < .001	d *=* 1.17

Note: Mean scores have been rescaled to vary between 1 and 7. CAT-Q = Camouflaging Autistic Traits Questionnaire. SF = Short Form.

**Table 8:** Correlations between the Full- and Short-Form CAT-Q in the Belcher et al. (2022) Sample.

	FF CAT-Q Total	FF Comp	FF Mask	FF Assim	CAT-Q SF	SF Comp	SF Mask	SF Assim
FF Comp	0.9 5**							
FF Mask	0.72**	0.60**						
FF Assim	0.84**	0.76**	0.31*					
CAT-Q SF	0.83**	0.90**	0.73**	0.79**				
SF Comp	0.72**	0.77**	0.59**	0.64**	0.90**			
SF Mask	0.62**	0.64**	0.79**	0.41**	0.82**	0.63**		
SF Assim	0.67**	0.71**	0.30*	0.84**	0.79**	0.60**	0.41**	
AQ	0.54**	0.58**	0.05	0.68**	0.47**	0.44**	0.14	0.62**

** p < .001;

* *p* < .01. Note: CAT-Q = Camouflaging Autistic Traits Questionnaire; FF = Full Form; SF = Short Form; Comp = Compensation subscale; Mask = Masking subscale; Assim = Assimilation subscale; AQ = Autism Quotient.

**Table 9:** Regression of AQ scores on Full-Form (FF) and Short-Form (SF) CAT-Q Subscales in the Belcher et al. (2022) Sample.

Predictor	R^2^ Adj	F(df)	B	p
FF Compensation	0.33	29.8 (1,78)	0.47	<0.001
FF Masking	−0.01	0.17 (1,78)	0.05	0.68
FF Assimilation	0.4 5	66.05 (1,78)	0.63	<0.001
SF Compensation	0.18	1876 (1,78)	0.90	<0.001
SF Masking	0.01	1.51 (1,78)	0.29	0.22
SF Assimilation	0.38	49.41 (1,78)	1.44	<0.001

## Data Availability

The data that support the findings of this study are available from the corresponding author, LH, upon reasonable request.
